# Expression Analysis of hsa-miR-181a-5p, hsa-miR-143-3p, hsa-miR-132-3p and hsa-miR-23a-3p as Biomarkers in Colorectal Cancer—Relationship to the Body Mass Index

**DOI:** 10.3390/cancers15133324

**Published:** 2023-06-24

**Authors:** Sofía Elena Tesolato, Daniel González-Gamo, Ana Barabash, Paula Claver, Sofía Cristina de la Serna, Inmaculada Domínguez-Serrano, Jana Dziakova, Carmen de Juan, Antonio José Torres, Pilar Iniesta

**Affiliations:** 1 Department of Biochemistry and Molecular Biology, Faculty of Pharmacy, Complutense University, 28040 Madrid, Spain; sofiteso@ucm.es (S.E.T.); daniel19@ucm.es (D.G.-G.); pclaver@ucm.es (P.C.); juchoca@ucm.es (C.d.J.); 2 Health Research Institute of the San Carlos Hospital (IdISSC), 28040 Madrid, Spain; ana.barabash@salud.madrid.org (A.B.); sdlsernae@gmail.com (S.C.d.l.S.); idserrano2@gmail.com (I.D.-S.); jana.dziakova@gmail.com (J.D.); antoniojose.torres@salud.madrid.org (A.J.T.); 3 CIBERDEM (Network Biomedical Research Center for Diabetes and Associated Metabolic Diseases), Carlos III Institute of Health, 28029 Madrid, Spain; 4 Endocrinology & Nutrition Service, San Carlos Hospital, 28040 Madrid, Spain; 5 Department of Medicine, Faculty of Medicine, Complutense University, 28040 Madrid, Spain; 6 Digestive Surgery Service, San Carlos Hospital, 28040 Madrid, Spain

**Keywords:** colorectal cancer, adipose tissue, miRNAs, serum biomarkers, obesity

## Abstract

**Simple Summary:**

Colorectal cancer (CRC) is one of the main tumor pathologies in our society considering its incidence and mortality. Various authors have linked the development of CRC with overweight and obesity. However, no molecular markers have been defined to connect both pathologies and that can be assessed in serum for diagnostic and/or prognostic purposes. The main objective of this work is to analyze and correlate the expression levels of four miRNAs previously associated with cancer and/or obesity in patients affected by CRC, as well as in a control group without cancer, considering the body mass index (BMI) of subjects. The main novelty of this study consists in the variety of samples investigated: adipose tissues, omental and subcutaneous; serum; and tumor and non-tumor tissues in the case of CRC patients. Results from this work allow us to conclude about the utility mainly of hsa-miR-143-3p and hsa-miR-181a-5p in the clinical management of CRC.

**Abstract:**

This work aims to investigate the expression levels of four preselected miRNAs previously linked to cancer and/or obesity, with the purpose of finding potential biomarkers in the clinical management of CRC developed by patients showing different BMI values. We analyzed samples from a total of 65 subjects: 43 affected by CRC and 22 without cancer. Serum and both subcutaneous and omental adipose tissues (SAT and OAT) were investigated, as well as tumor and non-tumor colorectal tissues in the case of the CRC patients. The relative expression (2^−∆∆Ct^) levels of 4 miRNAs (hsa-miR-181a-5p, hsa-miR-143-3p, has-miR-132-3p and hsa-miR-23a-3p) were measured by RT-qPCR. Serum, SAT and OAT expression levels of these miRNAs showed significant differences between subjects with and without CRC, especially in the group of overweight/obese subjects. In CRC, serum levels of hsa-miR-143-3p clearly correlated with their levels in both SAT and OAT, independently of the BMI group. Moreover, hsa-miR-181a-5p could be considered as a biomarker in CRC patients with BMI ≥ 25 Kg/m^2^ and emerges as a tumor location marker. We conclude that both adiposity and CRC induce changes in the expression of the miRNAs investigated, and hsa-miR-143-3p and hsa-miR-181a-5p expression analysis could be useful in the clinical management of CRC.

## 1. Introduction

Colorectal cancer (CRC) is still nowadays the third most common malignancy and the second cause of cancer-related mortality worldwide [1]. Primary tumor location conditions the molecular characteristics and the prognosis of CRC, being the most notable differences in right-colon cancers with respect to left-colon and rectal cancers. CRC development is believed to be a multi-step process involving both genetic and environmental factors, several of which are related to lifestyle [2]. Obesity is a known risk factor for CRC and is related to worse clinical outcomes, although the relationship between both diseases is yet not fully understood. Moreover, subcutaneous and visceral adiposity seem to affect the tumor process differently [3].

MicroRNAs (miRNAs) are small single-stranded noncoding RNAs that are involved in gene expression regulation mechanisms, mainly at the translational level [4]. Since their discovery, miRNAs have been related to several processes including cell proliferation or apoptosis, and their dysregulation has been linked to different types of cancer including CRC [5,6,7]. In addition, miRNAs are secreted to blood in exosomes or bound to lipoproteins, where they are particularly stable and resistant to ribonuclease degradation. Thus, circulating miRNAs detectable in serum or plasma are easily accessible potential biomarkers for CRC detection and the prediction of CRC prognosis [8]. Furthermore, recent studies relate adipogenesis and obesity with altered miRNA levels. However, the role of miRNAs as a possible link between obesity and cancer needs further investigation [9].

Herein, we present an expression study evaluating four miRNAs, namely, hsa-miR-181a-5p, hsa-miR-143-3p, hsa-miR-132-3p and hsa-miR-23a-3p, which have been previously related to CRC and/or obesity [10,11,12,13,14,15,16,17,18,19,20]. Particularly, hsa-miR-181a-5p was reported to target the tumor suppressor Wnt inhibitory factor 1 (WIF-1) and has been linked to tumor growth, liver metastasis and poorer overall survival in CRC [10,11] Moreover, hsa-miR-181a-5p was proven to promote angiogenesis in both in vitro and in vivo assays by indirectly activating the SRC kinase/Vascular Endothelial Growth Factor (SRC/VEGF) pathway [12]. Similarly, hsa-miR-23a-3p seems to enhance tumor and vascular growth in CRC via the Signal Transducer and Activator of Transcription 3/miR-23a-3p/Semaphorin 6D (STAT3/miR-23a-3p/SEMA6D) axis induced by Interleukin-17C (IL-17C), which also leads to VEGF production [13]. However, both miRNAs were found to be downregulated in adipose tissues from obese patients with respect to non-obese patients and were proven to have a role in insulin signaling [14]. On the other hand, hsa-miR-143-3p and hsa-miR-132-3p have been related to tumor suppression. As part of the hsa-miR-143/145 cluster, hsa-miR-143-3p is significantly decreased in CRC tissues and has oncostatic functions [15], as it prevents tumor growth and inhibits cancer metastasis [16,17]. The hsa-miR-132-3p downregulation correlates with tumor growth, invasion and metastasis [18]. With respect to obesity, hsa-miR-143-3p was positively correlated to insulin-mediated lipogenesis in subcutaneous adipose tissue [19] and induced insulin resistance in mouse models by inhibiting the AKT pathway [20].

Previous studies have shown that the measurement of miRNAs in the blood could complement current screening methods for CRC and might provide new insights into mechanisms of tumorigenesis and metastasis [21]. Additionally, hsa-miR-181a-5p and hsa-miR-143-3p circulating levels have been associated with therapy response in metastatic CRC [22]. Moreover, hsa-miR-23a-3p circulating levels were related to CRC diagnosis [23]. However, the differences between studies highlight the need to perform additional works that contribute to clarifying the role of miRNAs in the clinical management of the CRC patients.

Our work aims to analyze the expression levels of the four chosen miRNAs in serum and tissues from CRC subjects showing different BMI values, as well as in a control group without cancer, with the purpose of assessing their potential as biomarkers in CRC. To our knowledge, this is the first work that jointly evaluates the effect of both diseases, CRC and obesity, in the expression of selected miRNAs in human samples.

## 2. Materials and Methods

### 2.1. Patients and Samples

The population for the study included 43 colorectal cancer (CRC) patients who underwent a cancer-related surgery between 2021 and 2022, as well as 22 subjects without cancer (control group) who underwent other surgeries not related to this disease. In both cases, patients were operated on in the San Carlos Hospital (Madrid, Spain). Written informed consent was obtained from patients prior to investigation. In addition, written approval to develop this study was obtained from the Clinical Research Ethics Committee of the San Carlos Hospital (C.I. 19/549-E_BC, 27/12/2019), assuring the confidentiality of data to patients.

Age, gender and BMI values from the total of subjects considered, as well as CRC features (tumor location and TNM stage) are shown in Table 1.

Patients were classified according to their BMI values, following the criteria of the World Health Organization (WHO). Patients with BMI ≤ 24.9 Kg/m^2^ were defined as normal weight (11 cases: 8 CRC patients and 3 controls); and patients with BMI ≥ 25 kg/m^2^ were classified in the overweight/obesity group (54 cases, 35 with CRC and 19 controls). Recruitment was carried out with independence from gender, age of the patient or tumor stage, in the case of CRC subjects. No CRC patient had received chemo or radiotherapy before the surgery and inclusion in the study, and exclusion criteria included a previous digestive surgery, inflammatory diseases and antibiotic treatment one month before the surgical intervention. The control group without cancer included subjects undergoing surgeries not related to the pathologies investigated in this work (cholelithiasis or hiatus hernia) and who did not have a family history of cancer.

A total number of 195 samples of serum and paired abdominal subcutaneous and omental adipose tissue (SAT and OAT, respectively) were collected prospectively from both groups of patients included in the study. Moreover, in the case of CRC subjects, tumor and paired non-tumor colorectal tissue samples were also obtained. For serum samples, blood was collected the day of the surgery after an overnight fast and centrifuged within 10 min to 1 h after the extraction (10 min and 1300× *g* of speed). The serum was then separated and stored at −80 °C until analysis. Adipose tissue samples were collected during the surgery and immediately submerged in 1 mL of RNAlater RNA Stabilization Reagent (Qiagen, Hilden, Germany), stored 24 h at 4 °C and subsequently frozen to −80 °C until processing. Finally, all colorectal tissues from CRC patients were provided by the San Carlos Hospital Biobank (B.0000725) via the project PT2020/00074, subsidized by the Carlos III Institute of Health (ISCIII) and co-funded by the European Union through the European Regional Development Fund (ERDF). The said Biobank belongs to the San Carlos Health Research Institute (IdISSC) and is part of the national network of Biobanks. After their extraction during surgery, colorectal tissue samples were instantly embedded in Tissue-Tek OCT and frozen in liquid nitrogen at −80 °C. Tumor samples were cryostat sectioned, H&E stained and examined microscopically by two independent pathologists to confirm the presence of ≥80% tumor cells. Paired non-tumor samples from the same patients were obtained at the greatest possible distance from the tumor area (≥10 cm, if possible) and microscopically confirmed. CRC staging was carried out following the NCCN guidelines (National Comprehensive Cancer Network) v 2.2022.

### 2.2. RNA Extraction and microRNA (miRNA) Expression Analysis

Total RNA in serum samples was extracted using the miRNeasy Serum/Plasma Advanced Kit (Qiagen), following the manufacturer’s protocol, from a starting volume of 200 μL per sample and eluting in 20 μL of RNase Free Water (RFW). RNA recovery was increased by adding 1 μg of MS2 carrier RNA (Roche) to the sample before the extraction. Adipose tissues were cut and washed in 1 mL PBS 1x to remove the RNAlater, and briefly homogenized in 700 μL of QIAzol™ Lysis Reagent (Qiagen) using the Ultra-Turrax™ homogenizer. Next, total RNA extraction was performed using the miRNeasy Mini Kit (Qiagen), according to the manufacturer’s instructions and with a final elution volume of 30 μL of RFW. To optimize the extraction, 5PRIME Phase Lock Gel (PLG) heavy tubes (VWR International Eurolab, Barcelona, Spain) were used during the phase separation step. OCT from colorectal tissue sections was also removed by washing with 1 mL PBS 1x and centrifuging at 3000 rpm during 5–10 min at room temperature, prior to homogenization in 700 μL of QIAzol™ Lysis Reagent and RNA extraction following the same protocol as for adipose tissues.

After the extraction, a reverse transcription reaction was performed in all the samples to convert total RNA into cDNA, by using the miRCURY^®^ LNA^®^ RT Kit (Qiagen) for a final reaction volume of 10 μL per sample. Following the kit’s protocol, 10 ng of template RNA from each tissue sample was used for the reaction. For serum samples, 2 μL of the extracted RNA was used directly. The incubation was performed in the Applied Biosystems™ Veriti™ 96-Well Thermal Cycler (Thermo Fisher Scientific, Madrid, Spain). Afterwards, cDNA was stored at −20 °C until use.

miRNA expression was determined by real-time quantitative PCR (qPCR), using the miRCURY LNA SYBR^®^ Green PCR Kit (Qiagen) as well as an individual miRCURY LNA miRNA PCR Assay (Qiagen) for each of the four miRNAs analyzed (hsa-miR-181a-5p, hsa-miR-143-3p, hsa-miR-132-3p and hsa-miR-23a-3p) and for hsa-miR-103-3p, used as an internal control. This miRNA has been extensively described in the literature as a reference miRNA in expression studies, both in tumor and adipose tissues and in serum from patients with and without cancer [24,25,26]. Expression studies, both in serum and tissue samples from CRC and control subjects, showed that the coefficient of variation for hsa-miR-103-3p was lower than 5% considering the Ct values obtained in the RT-qPCR experiments. Reactions were performed in 0.1 mL MicroAmp^®^ Fast Optical 96-Well Reaction Plates. Prior to the PCR reaction, cDNA was diluted to 1:60 (for tissue samples) or to 1:30 (for serum samples). Reaction setup was performed for a 10 μL/well reaction, and ROX reference dye was added as a 20x concentrate (0.5 μL per well) as recommended by the manufacter for a StepOnePlus™ Real-Time PCR System (Thermo Fisher Scientific). The relative expression (RQ) calculation was carried out following the 2^−ΔΔCt^ method.

### 2.3. Statistical Analysis

Statistical analyses were performed using the IBM^®^ SPSS^®^ Statistics software package version 27 (IBM Inc., New York, NY, USA). The normality of the data was assessed using the Shapiro–Wilk (n < 50) or Kolmogorov–Smirnov (n ≥ 50) tests, and Levene’s test for equality of variances was used to analyze the homoscedasticity conditions of the variables. The Chi-square test was employed to compare categorical variables. Correlations between quantitative variables were established with Pearson (parametric variables) and Spearman (non-parametric variables) tests. To compare the means of two related variables, the Wilcoxon signed-rank test was performed. Finally, the mean values of the quantitative data between two or more study groups were compared using either parametric tests (Student’s *t* test for 2 categories and ANOVA for 3 or more categories) or non-parametric tests (Mann–Whitney U test for 2 categories and Kruskal–Wallis test for 3 or more categories). In any case, *p* values < 0.05 were considered statistically significant.

## 3. Results

### 3.1. Differences in miRNA Expression in Serum and Adipose Tissues between Subjects with and without CRC. Relationship to the BMI Values

Table 2 shows the differences in mean serum and both SAT and OAT levels of the four miRNAs analyzed (hsa-miR-181a-5p, hsa-miR-143-3p, hsa-miR-132-3p and hsa-miR-23a-3p) between CRC patients and control subjects. As can be observed, most of the adipose tissue expressions were higher in the control group with respect to the CRC cases. Particularly, OAT expression was significantly diminished in patients with CRC for three of the four miRNAs studied (hsa-miR-181a-5p, *p* < 0.001, hsa-miR-143-3p, *p* = 0.031, and hsa-miR-23a-3p, *p* < 0.001), whereas SAT expression was higher in controls for hsa-miR-132-3p (*p* = 0.005) and hsa-miR-23a-3p (*p* = 0.044).

In Table 3, we show the mean miRNA expression levels in relation to the BMI of subjects. Thus, regarding the groups of normal weight (BMI ≤ 24.9 kg/m^2^) and overweight/obese (BMI ≥ 25 kg/m^2^), the main differences were found between CRC and controls from the overweight/obese group. Specifically, the four miRNA analyzed in this work were significantly higher in OAT from subjects without cancer showing BMI values ≥ 25 kg/m^2^. Also, hsa-miR-132-3p SAT expression was still significantly diminished in the overweight/obese CRC group (*p* = 0.008). However, in the group of normal weight, the differences in miRNA expression levels were not as evident and did not follow a specific profile. Significantly higher levels in serum were only observed for hsa-miR-181a-5p and hsa-miR-23a-3p in the normal weight group without cancer (*p* = 0.008 and *p* = 0.034, respectively).

Only considering CRC patients, our data indicated remarkable differences in miRNA serum expression levels in relation to the BMI values (Table 4). More specifically, serum levels of the miRNAs studied in this work increased in overweight/obese CRC patients with respect to the normal weight group. These differences could be noticed in the four miRNAs analyzed, although they were only significant for hsa-miR-181a-5p (*p* = 0.045), and were borderline-significant for hsa-miR-143-3p (*p* = 0.054), hsa-miR-132-3p (*p* = 0.088) and hsa-miR-23a-3p (*p* = 0.054).

Serum miRNA expression levels in subjects without cancer (control group) with different BMIs did not report significant differences (Table 5), although a downward trend was observed for most of the miRNA included in the study. Therefore, in the control group, the trend was the opposite of that detected in the CRC cases, with a decrease in miRNAs serum levels in the group of overweight/obese patients without cancer. However, it is necessary to consider that the normal weight group within the controls consists of only three cases. For this reason, Table 5 shows, in addition to the means and the standard errors, the values of the medians for each of the groups being compared.

### 3.2. Correlations between Serum and Adipose Tissue miRNA Expression in Subjects with and without CRC

**Figure 1:**
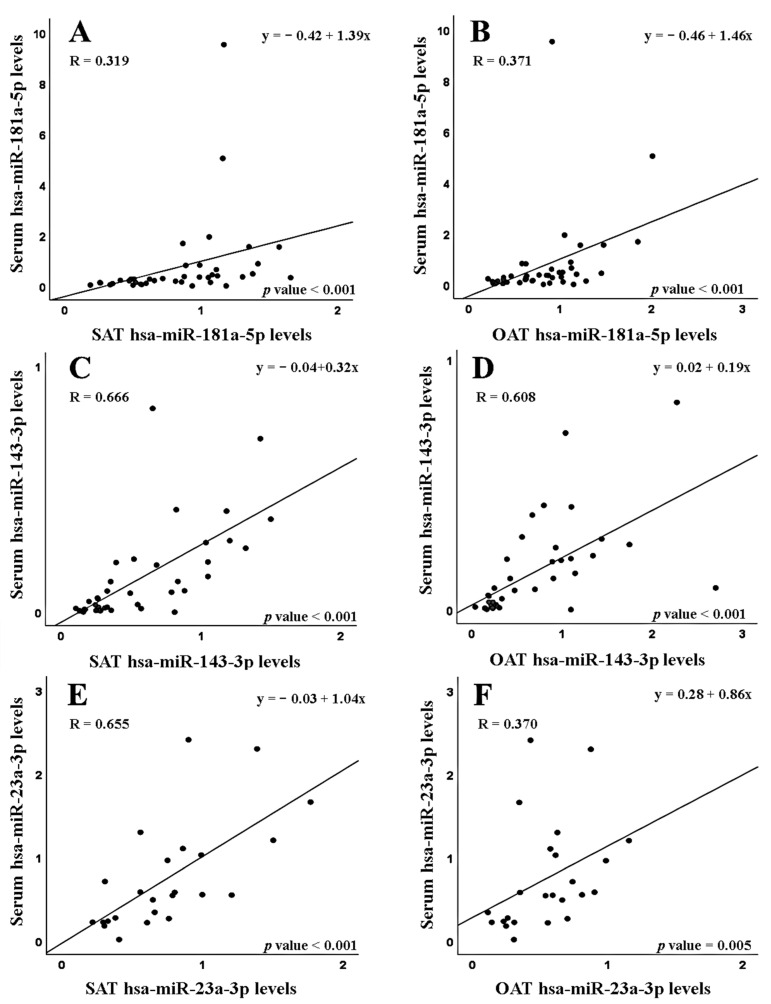
Correlations (Spearman test) between miRNA levels in serum and adipose tissues (subcutaneous, SAT; and omental, OAT) of colorectal cancer patients, for hsa-miR-181a-5p (**A**,**B**), hsa-miR-143-3p (**C**,**D**) and hsa-miR-23a-3p (**E**,**F**).

Linear regression analysis, comparing serum and tissue miRNA levels in patients with CRC, showed relevant correlations in the case of hsa-miR-143-3p levels in serum and its levels in both SAT and OAT (R = 0.666 and R = 0.608, respectively; *p* < 0.001 in both cases). Moreover, a clear correlation was detected between hsa-miR-23a-3p levels in serum and its levels in SAT (R = 0.655, *p* < 0.001), and a less relevant correlation between serum and OAT hsa-miR-23a-3p expression levels (R = 0.370, *p* = 0.005) (Figure 1). As it is also shown in Figure 1, significant associations were detected between the serum levels of hsa-miR-181a-5p and the expression of this miRNA in SAT and OAT; however, in these cases no relevant correlations were found (R = 0.319 and R = 0.371, respectively; *p* < 0.001 in both cases).

When patients were divided according to their BMI (Table 6), both serum–SAT and serum–OAT correlations kept in the two BMI groups were considered in this work for hsa-miR-143-3p (serum–SAT correlation: *p* = 0.004 in normal weight patients, *p* < 0.001 in overweight/obese patients; serum–OAT correlation: *p* = 0.028 in normal weight patients and *p* < 0.001 in overweight/obese patients). For hsa-miR-181a-5p, no significant correlations were detected in the group of the normal weight patients and were only obtained when correlations were established in the group of cases showing BMI values ≥ 25 kg/m^2^ (*p* < 0.001 for serum–SAT and serum–OAT correlations). For hsa-miR-132-3p, serum–SAT and serum–OAT correlations were significant just in the normal weight group of patients (*p* = 0.047 and *p* = 0.045, respectively). Finally, for hsa-miR-23a-3p, only a serum–SAT correlation in overweight/obese patients was found (*p* = 0.002).

**Figure 2:**
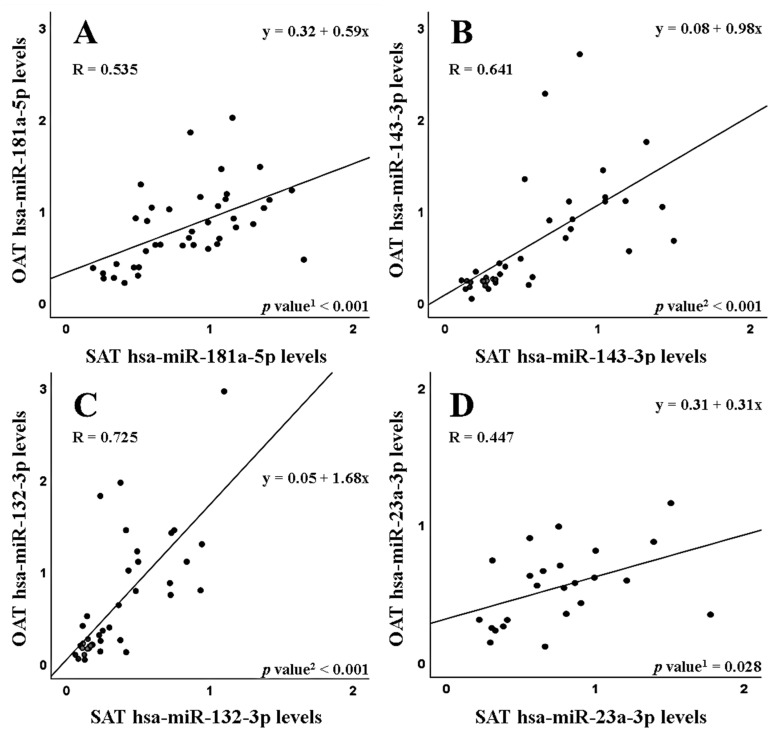
Correlations in miRNA levels between both adipose tissues (subcutaneous and omental) of colorectal cancer patients, for hsa-miR-181a-5p (**A**), hsa-miR-143-3p (**B**), hsa-miR-132-3p (**C**) and hsa-miR-23a-3p (**D**). ^1^ Pearson’s correlation test; ^2^ Spearman’s correlation test.

Besides the correlation with serum, there was a positive correlation between the expression levels of both adipose tissues (SAT and OAT) in CRC patients, which was significant for the four miRNAs analyzed (Figure 2, *p* < 0.001 for hsa-miR-181a-5p, hsa-miR-143-3p and hsa-miR-132-3p, *p* = 0.028 for hsa-miR-23a-3p). When patients were divided according to their BMI values (Table 7), the SAT–OAT correlation remained in the two groups for hsa-miR-143-3p (*p* = 0.004 in normal weight, *p* < 0.001 in overweight/obese) and for hsa-miR-132-3p (*p* = 0.010 in normal weight, *p* < 0.001 in overweight/obese). For hsa-miR-181a-5p, the SAT–OAT correlation disappeared in normal weight CRC patients and was significant only in the group of overweight/obese cases (*p* < 0.001).

Interestingly, all the correlations indicated in this section for the group of patients affected by CRC were not observed in the control cases without cancer.

### 3.3. miRNA Expression Levels in Tumor and Non-Tumor Tissues from CRC Patients—Differences in Relation to Tumor Location

Relative expression levels of the studied miRNAs were compared between tumor and paired non-tumor tissues from CRC patients (Table 8). Levels of hsa-miR-143-3p, hsa-miR-132-3p and hsa-miR-23a-3p were significantly lower in colorectal tumor samples with respect to their matched non-tumor tissues (*p* < 0.001 for hsa-miR-143-3p, *p* = 0.031 for hsa-miR-132-3p and *p* = 0.021 for hsa-miR-23a-3p).

Comparison of the mean miRNA expression levels between samples from CRC patients with different primary tumor location revealed important differences, most of them found between right- and left-colon cancer patients (Table 9). More particularly, the tumor/non tumor expression ratio for hsa-miR-181a-5p was significantly lower in right-colon cancers compared to left-colon cancers (*p* = 0.002). Interestingly, this was also found when right-colon cancers were compared to rectal cancers (mean T/N ratio ± standard error: 0.58 ± 0.076 in right-colon cancers and 1.02 ± 0.134 in rectal cancers, *p* = 0.019 in Students’ *t* test). On the other hand, non-tumor tissue expression levels were higher in right-colon cancers than in left-colon cancers for hsa-miR-181a-5p, hsa-miR-132-3p and hsa-miR-23a-3p (*p* = 0.044, *p* = 0.028 and *p* = 0.016, respectively). There were also differences in the adipose tissue levels of hsa-miR-23a-3p: both SAT and OAT expressions from right-sided CRC patients were significantly increased with respect to the ones from left-sided CRC patients (*p* = 0.003 and *p* = 0.011, respectively).

## 4. Discussion

In this study, we analyzed the expression levels of four miRNAs (hsa-miR-181a-5p, hsa-miR-143-3p, hsa-miR-132-3p and hsa-miR-23a-3p), previously related to cancer and/or obesity, in serum and tissues from subjects with and without CRC. Our results show noticeable differences between the subjects affected by CRC and the control group without cancer, as well as in relation to the BMI values of the subjects and tumor location.

Comparing the relative miRNA expressions with relation to the cancer process, adipose tissues (SAT and OAT) from the subjects affected by CRC had diminished miRNA levels with respect to the ones from the control group, particularly OAT. Most of the differences were preserved when patients with BMI values ≥ 25 kg/m^2^ (either overweight or obese) were considered. Other studies highlighted the role of different miRNAs in favoring different diseases, such as cancer or metabolic processes, allowing the authors to conclude about the possible pathophysiological role of miRNAs. Thus, hsa-miR-132-3p levels were found to be decreased in serum and omental adipose tissue from obese patients with respect to non-obese individuals, and its presence in omental adipose tissue was negatively correlated to the visceral fat area and macrophage infiltration [27,28]. The expression of miRNAs in different tissues has been related to metabolic disorders such as obesity. One of the most relevant findings in the field of miRNA research in recent years is the discovery that miRNAs are packaged within cell-secreted exosomes; according to the authors, this fact could be critical for the crosstalk between different organs [29].

When the levels of the studied miRNAs were compared in CRC patients with relation to the BMI, serum levels of the four miRNAs were increased in the patients with higher BMI values (overweight or obese) with respect to the group of normal weight. These differences were statistically significant in the case of hsa-miR-181a-5p, and bordered significance for the other three miRNAs studied (hsa-miR-143-3p, hsa-miR-132-3p and hsa-miR-23a-3p). As previously mentioned, hsa-miR-181a-5p has been involved in CRC, mainly playing an oncogenic role [5]. Its levels in the colon cancer cells have been related to a metabolic shift from oxidative to glycolytic [30], as well as to increased tumor growth and to both angiogenic and metastatic properties [10,11,12]. Conversely, obesity has been linked to a reduction in the adipose tissue levels of this miRNA [14], so both diseases could be affecting hsa-miR-181a-5p expression in different directions. The miRNAs as an interplay between obesity and CRC are still poorly studied. Moreover, little is known about what happens to the circulating miRNA levels in the context of these two pathologies [31]. Given the results shown in our study, the increment of hsa-miR-181a-5p levels in the serum of overweight and obese CRC patients probably reflects the joint deregulation that both adiposity and the cancer process cause on the expression of this molecule in the tissues, giving a clue to the molecular interactions between both conditions and their impact on cancer prognosis.

In CRC patients, circulating levels of hsa-miR-143-3p and hsa-miR-23a-3p, detected in serum, were positively correlated to the levels detected in adipose tissues, both SAT and OAT. Thomou et al. found that adipose tissue dysfunction resulted in a significant decrease in blood exosomal miRNAs, demonstrating a considerable contribution of this type of tissue to the pool of circulating miRNAs that can exert their effects in distant locations [32]. In line with these findings, the miRNAs detected in the serum of the patients included in our study seem to be mainly secreted by the adipose tissue, and they may have an effect in the tumor microenvironment of CRC. Moreover, when our CRC patients were evaluated separately according to their BMI, some serum–adipose tissue correlations, particularly the ones for hsa-miR-181a-5p, were only present in patients with overweight or obesity, suggesting an influence of BMI on the relationship between both miRNA levels. On the other hand, the four miRNAs analyzed showed a positive correlation between their expressions in SAT and OAT from CRC patients, which were maintained in the two BMI groups for hsa-miR-143-3p and hsa-miR-132-3p but only in patients with BMI ≥ 25 kg/m^2^ in the case of hsa-miR-181a-5p. Although there is accumulating evidence supporting that subcutaneous and visceral adipose tissues have different metabolic and inflammatory characteristics and expression profiles [33,34], both fat depots show coordinated molecular adaptations to obesity and metabolic syndrome [35]. Circulating miRNAs secreted from different tissues could affect the metabolic profiles of distant organs, thereby facilitating metabolic organ crosstalk [36]. Therefore, it is possible that the expression of certain miRNAs is connected between both adipose tissues and related to the cancer process. Also in CRC patients, tumor tissues showed significantly lower expressions of hsa-miR-143-3p, hsa-miR-132-3p and hsa-miR-23a-3p with respect to their paired non-tumor tissues. Cancer has been associated with a global defect in miRNA production, possibly due to genetic or epigenetic changes affecting the miRNA genes, or because of alterations in some components of the miRNA biogenesis pathway such as Drosha or Dicer [5,37]. Moreover, matching the results shown in this study, hsa-miR-143-3p has previously been reported to be downregulated in tumor samples with respect to marginal normal mucosa of CRC patients [38], and hsa-miR-132-3p levels were found to be lower in CRC cell lines with respect to normal colonic cells, as well as in CRC tumor samples when compared to their paired normal tissue [18,39,40]. 

Finally, several studies have already mentioned differences in the miRNA expression profiles between CRCs with different primary tumor location [41,42,43], also showing a site-specific impact of these molecules on cancer survival [44]. Our results indicated that right-colon cancers had a lower tumor/non-tumor expression ratio for hsa-miR-181a-5p when compared to left-colon and rectal cancers. Although tumor levels alone for this miRNA were not significantly different between cancer sites, non-tumor tissue levels were significantly higher in the right colon. Thus, a decreased T/N ratio better reflects the reduction of hsa-miR-181a-5p in cancer cells with respect to their original tissue, which could be linked to the distinctive molecular nature of the right-sided CRCs. It seems relevant to highlight, as described in the introduction of this work, that functional studies carried out in CRC models have related the expression of hsa-miRNA-181a-5p with tumor growth and the promotion of angiogenesis and metastasis [10,12]. Furthermore, in the case of hsa-miR-23a-3p, the levels in both SAT and OAT were increased in patients in whom the cancer was located in the right colon, with respect to the ones in individuals with left-colon cancer. Recent evidence supports a crosstalk between adipose tissue and cancer cells, through which cancer cells may induce an ‘activated’ phenotype in the surrounding adipocytes that in turn secrete several molecules, including some miRNAs, capable of promoting tumor development and metastasis [45]. Stromal cells from the visceral adipose tissue neighboring colorectal tumors were proved to promote vasculogenesis and metastasis in obese CRC patients [46]. Specifically, hsa-miR-23a/b can be transferred through exosomes from adipocytes to hepatocellular carcinoma cells, where they increase cancer growth and chemoresistance [47]. In this context and given the oncogenic role of hsa-miR-23a-3p in CRC, such as it has been demonstrated through functional studies [13], increased adipose tissue levels of this miRNA in right-colon cancers could be indirectly related to the cancer characteristics and even drug resistance.

As the main limitation of this work, we should note the heterogeneity in age and gender of the populations under study. However, we consider that the results obtained in the present work, with a wide variety of samples analyzed, contribute to the advancement in the knowledge of the possible usefulness of miRNAs as biomarkers in CRC.

## 5. Conclusions

We highlight three main conclusions from the results of this work. Firstly, the four miRNAs (hsa-miR-181a-5p, hsa-miR-143-3p, hsa-miR-132-3p and hsa-miR-23a-3p) investigated appear to be deregulated in CRC. Secondly, considering serum–adipose tissue correlations for hsa-miR-143-3p, this miRNA could constitute a potential biomarker in CRC diagnosis, independently of the BMI group considered. Finally, hsa-miR-181a-5p emerges as a tumor location marker that could reflect the molecular characteristics associated with CRCs from different colon sides and the rectum. Once the results are validated in populations with a larger number of cases, mainly hsa-miR-181a-5p expression analysis could be useful in the clinical management of CRC, primarily as a tumor location biomarker.

## Figures and Tables

**Table 1:** Clinico-pathological features of subjects with and without colorectal cancer.

Variable	CRC * Group (N = 43 Patients)	Control Group (N = 22 Patients)	*p* Value
Mean age (years) ± standard error	71.65 ± 1.91	55.50 ± 2.87	<0.001 ^1^
Gender, N (%)			0.017 ^2^
Male	29 (67.44)	8 (36.36)	
Female	14 (32.56)	14 (63.64)	
BMI ^≠^ group, N (value, mean ± standard error)			0.613 ^2^
Normal weight (BMI ^≠^ ≤ 24.9 Kg/m^2^)	8 (23.02 ± 0.62)	3 (23.55 ± 0.53)	
Overweight/Obesity (BMI ^≠^ ≥ 25 kg/m^2^)	35 (27.72 ± 0.52)	19 (37.89 ± 1.78)	
Tumor location, N (%)			
Right colon	23 (53.49)	-	
Left colon	13 (30.23)	-	
Rectum	7 (16.28)	-	
TNM stage, N (%)			
I	6 (13.95)	-	
II	16 (37.21)	-	
III	18 (41.86)	-	
IV	3 (6.98)	-	

* Colorectal cancer; ^≠^ body mass index; ^1^ Student’s *t* test; ^2^ Chi-square test.

**Table 2:** Mean relative miRNA expression in colorectal cancer and control subjects.

	Mean Relative miRNA Expression (2^−∆∆Ct^) ± Standard Error		
miRNA Samples	CRC ^3^	Controls	*p* Value ^4^
hsa-miR-181a-5p			
Serum	0.23 ± 0.059	0.25 ± 0.037	0.128
SAT ^1^	0.66 ± 0.073	0.84 ± 0.164	0.365
OAT ^2^	0.63 ± 0.067	1.25 ± 0.232	<0.001
hsa-miR-143-3p			
Serum	0.04 ± 0.009	0.02 ± 0.003	0.380
SAT ^1^	0.29 ± 0.025	0.39 ± 0.110	0.840
OAT ^2^	0.24 ± 0.019	0.46 ± 0.105	0.031
hsa-miR-132-3p			
Serum	0.18 ± 0.031	0.18 ± 0.078	0.482
SAT ^1^	0.18 ± 0.018	0.44 ± 0.139	0.005
OAT ^2^	0.22 ± 0.023	0.49 ± 0.165	0.119
hsa-miR-23a-3p			
Serum	0.74 ± 0.126	1.21 ± 0.453	0.242
SAT ^1^	0.75 ± 0.083	1.99 ± 0.824	0.044
OAT ^2^	0.54 ± 0.057	1.42 ± 0.353	<0.001

^1^ Subcutaneous adipose tissue; ^2^ omental adipose tissue; ^3^ colorectal cancer; ^4^ Mann–Whitney U test.

**Table 3:** Mean relative miRNA expression comparison between colorectal cancer patients and controls according to the body mass index.

	Mean Relative miRNA Expression (2^−∆∆Ct^) ± Standard Error					
	Normal Weight		*p* Value ^4,5^	Overweight/Obese		*p* Value ^4,5^
miRNA Samples	CRC ^3^	Control		CRC ^3^	Control	
hsa-miR-181a-5p						
Serum	0.08 ± 0.028	0.29 ± 0.041	0.008 ^4^	0.26 ± 0.068	0.24 ± 0.046	0.677 ^5^
SAT ^1^	0.63 ± 0.078	0.70 ± 0.097	0.602 ^4^	0.67 ± 0.087	0.87 ± 0.197	0.403 ^5^
OAT ^2^	0.73 ± 0.161	0.86 ± 0.157	0.640 ^4^	0.61 ± 0.075	1.33 ± 0.272	<0.001 ^5^
hsa-miR-143-3p						
Serum	0.01 ± 0.004	0.03 ± 0.010	0.117 ^4^	0.04 ± 0.011	0.01 ± 0.002	0.071 ^5^
SAT ^1^	0.25 ± 0.056	0.36 ± 0.102	0.340 ^4^	0.29 ± 0.028	0.40 ± 0.132	0.538 ^5^
OAT ^2^	0.23 ± 0.029	0.30 ± 0.088	0.371 ^4^	0.25 ± 0.022	0.49 ± 0.122	0.032 ^5^
hsa-miR-132-3p						
Serum	0.08 ± 0.025	0.46 ± 0.361	0.289 ^5^	0.20 ± 0.036	0.10 ± 0.013	0.137 ^5^
SAT ^1^	0.15 ± 0.031	0.22 ± 0.072	0.315 ^4^	0.18 ± 0.021	0.48 ± 0.164	0.008 ^5^
OAT ^2^	0.21 ± 0.013	0.15 ± 0.025	0.054 ^4^	0.22 ± 0.028	0.55 ± 0.190	0.043 ^5^
hsa-miR-23a-3p						
Serum	0.26 ± 0.111	3.10 ± 1.921	0.034 ^5^	0.83 ± 0.141	0.70 ± 0.119	0.921 ^5^
SAT ^1^	0.50 ± 0.171	1.09 ± 0.024	0.086 ^4^	0.80 ± 0.091	2.17 ± 0.987	0.187 ^5^
OAT ^2^	0.42 ± 0.130	0.79 ± 0.075	0.141 ^4^	0.57 ± 0.063	1.54 ± 0.410	0.002 ^5^

^1^ Subcutaneous adipose tissue; ^2^ omental adipose tissue; ^3^ colorectal cancer; ^4^ Student’s *t* test; ^5^ Mann–Whitney U test.

**Table 4:** Mean miRNA levels in serum of colorectal cancer patients according to their body mass index.

	Serum Mean Relative miRNA Expression (2^−∆∆Ct^) ± Standard Error		
miRNA	Normal Weight CRC ^1^ Patients	Overweight/obese CRC ^1^ Patients	*p* Value ^2^
hsa-miR-181a-5p	0.08 ± 0.028	0.26 ± 0.068	0.045
hsa-miR-143-3p	0.01 ± 0.004	0.04 ± 0.011	0.054
hsa-miR-132-3p	0.08 ± 0.025	0.20 ± 0.036	0.088
hsa-miR-23a-3p	0.26 ± 0.111	0.83 ± 0.141	0.054

^1^ Colorectal cancer; ^2^ Mann–Whitney U test.

**Table 5:** Mean miRNA levels in serum of control subjects according to their body mass index.

	Serum Mean (and Median) Relative miRNA Expression (2^−∆∆Ct^) ± Standard Error		
miRNA	Normal Weight Controls	Overweight/Obese Controls	*p* Value ^1^
hsa-miR-181a-5p	0.29 ± 0.041 (0.252)	0.24 ± 0.046 (0.175)	0.073
hsa-miR-143-3p	0.03 ± 0.010 (0.017)	0.01 ± 0.002 (0.013)	0.052
hsa-miR-132-3p	0.46 ± 0.361 (0.117)	0.10 ± 0.013 (0.089)	0.243
hsa-miR-23a-3p	3.10 ± 1.921 (1.820)	0.70 ± 0.119 (0.571)	0.073

^1^ Mann–Whitney U test.

**Table 6:** Correlations between miRNA levels in serum and adipose tissues (subcutaneous and omental) of colorectal cancer patients.

	Serum–Adipose Tissue Correlations in miRNA Levels Correlation Coefficients (*p* Values)							
	hsa-miR-181a-5p		hsa-miR-143-3p		hsa-miR-132-3p		hsa-miR-23a-3p	
BMI ^1^ Group	S ^2^—SAT ^3^	S ^2^—OAT ^4^	S ^2^—SAT ^3^	S ^2^—OAT ^4^	S ^2^—SAT ^3^	S ^2^—OAT ^4^	S ^2^—SAT ^3^	S ^2^—OAT ^4^
Normal weight	0.619 ^5^ (0.102 ^5^)	0.190 ^5^ (0.651 ^5^)	0.881 ^5^ (0.004 ^5^)	0.762 ^5^ (0.028 ^5^)	0.714 ^5^ (0.047 ^5^)	0.717 ^6^ (0.045 ^6^)	0.782 ^6^ (0.218 ^6^)	0.400 ^5^ (0.600 ^5^)
Overweight/ Obese	0.646 ^5^ (<0.001 ^5^)	0.665 ^5^ (<0.001 ^5^)	0.708 ^5^ (<0.001 ^5^)	0.740 ^5^ (<0.001 ^5^)	−0.097 ^5^ (0.599 ^5^)	−0.192 ^5^ (0.277 ^5^)	0.657 ^5^ (0.002 ^5^)	0.430 ^5^ (0.058 ^5^)

^1^ Body mass index; ^2^ serum; ^3^ subcutaneous adipose tissue; ^4^ omental adipose tissue; ^5^ Spearman’s correlation test; ^6^ Pearson’s correlation test.

**Table 7:** Correlations in miRNA levels between both adipose tissues (subcutaneous and omental) of colorectal cancer patients.

	SAT ^1^–OAT ^2^ Correlations in miRNA Expression Correlation Coefficients (*p* Values)			
BMI ^3^ Group	hsa-miR-181a-5p	hsa-miR-143-3p	hsa-miR-132-3p	hsa-miR-23a-3p
Normal weight	0.636 ^4^ (0.090 ^4^)	0.881 ^5^ (0.004 ^5^)	0.833 ^5^ (0.010 ^5^)	0.400 ^5^ (0.600 ^5^)
Overweight/Obese	0.560 ^4^ (<0.001 ^4^)	0.792 ^5^ (<0.001 ^5^)	0.755 ^5^ (<0.001 ^5^)	0.350 ^4^ (0.131 ^4^)

^1^ Subcutaneous adipose tissue; ^2^ omental adipose tissue; ^3^ body mass index; ^4^ Pearson’s correlation test; ^5^ Spearman’s correlation test.

**Table 8:** Comparison of miRNA levels between tumor and paired non-tumor colorectal tissues of colorectal cancer patients.

	Mean Relative miRNA Expression (2^−∆∆Ct^) ± Standard Error		
miRNA	Tumor Tissue	Non-Tumor Tissue	*p* Value ^1^
hsa-miR-181a-5p	1.02 ± 0.092	1.26 ± 0.137	0.278
hsa-miR-143-3p	0.59 ± 0.152	2.30 ± 0.456	<0.001
hsa-miR-132-3p	0.97 ± 0.124	1.38 ± 0.164	0.031
hsa-miR-23a-3p	1.17 ± 0.221	1.46 ± 0.176	0.021

^1^ Wilcoxon signed-rank test.

**Table 9:** Comparison of the mean miRNA levels between tumors from the right colon and the left colon.

	Mean Relative miRNA Expression (2^−∆∆Ct^) ± Standard Error		
miRNA Samples	Tumors from the Right Colon	Tumors from the Left Colon	*p* Value ^4,5^
hsa-miR-181a-5p			
Tumor tissue	0.88 ± 0.136	1.30 ± 0.242	0.119 ^4^
Non-tumor tissue	1.61 ± 0.192	0.96 ± 0.210	0.044 ^4^
T/N ^1^	0.58 ± 0.076	1.64 ± 0.331	0.002 ^5^
Serum	0.19 ± 0.034	0.16 ± 0.032	0.536 ^4^
SAT ^2^	0.78 ± 0.124	0.53 ± 0.063	0.133 ^4^
OAT ^3^	0.66 ± 0.097	0.58 ± 0.093	0.591 ^4^
hsa-miR-143-3p			
Tumor tissue	0.48 ± 0.244	0.67 ± 0.320	0.673 ^5^
Non-tumor tissue	2.51 ± 0.948	2.46 ± 1.118	0.800 ^5^
T/N ^1^	2.36 ± 2.233	2.46 ± 2.045	0.735 ^5^
Serum	0.05 ± 0.017	0.01 ± 0.002	0.105 ^5^
SAT ^2^	0.33 ± 0.042	0.23 ± 0.029	0.100 ^4^
OAT ^3^	0.27 ± 0.026	0.21 ± 0.026	0.161 ^4^
hsa-miR-132-3p			
Tumor tissue	0.98 ± 0.285	0.94 ± 0.277	0.800 ^5^
Non-tumor tissue	1.81 ± 0.350	0.79 ± 0.139	0.028 ^5^
T/N ^1^	1.20 ± 0.749	1.57 ± 0.731	0.272 ^5^
Serum	0.21 ± 0.043	0.16 ± 0.071	0.247 ^5^
SAT ^2^	0.22 ± 0.034	0.13 ± 0.008	0.083 ^5^
OAT ^3^	0.26 ± 0.037	0.18 ± 0.038	0.133 ^4^
hsa-miR-23a-3p			
Tumor tissue	1.38 ± 0.387	0.83 ± 0.160	0.398 ^5^
Non-tumor tissue	1.85 ± 0.249	0.89 ± 0.189	0.016 ^4^
T/N ^1^	0.87 ± 0.316	1.36 ± 0.578	0.205 ^5^
Serum	0.92 ± 0.175	0.47 ± 0.134	0.054 ^5^
SAT ^2^	1.01 ± 0.127	0.44 ± 0.071	0.003 ^4^
OAT ^3^	0.71 ± 0.079	0.38 ± 0.079	0.011 ^4^

^1^ Tumor/non-tumor tissue expression ratio; ^2^ subcutaneous adipose tissue; ^3^ omental adipose tissue; ^4^ Student’s *t* test; ^5^ Mann–Whitney U test.

## Data Availability

The datasets used and/or analyzed during the current study are contained within the article.
